# Seagrasses in hot water: mapping thermal risk and resilience in a warming ocean

**DOI:** 10.1111/nph.70903

**Published:** 2026-01-28

**Authors:** Cloverley M. Lawrence

**Affiliations:** ^1^ South African National Parks, Rondevlei Scientific Services Sedgefield 6573 South Africa; ^2^ Institute for Coastal and Marine Research Nelson Mandela University Gqeberha 6031 South Africa

**Keywords:** climate adaptation planning, daily carbon balance, foundation species resilience, spatial risk mapping, temperature–performance relationships

## Abstract

This article is a Commentary on Said *et al*. (2026), **249**: 2835–2851.

Marine heatwaves are no longer rare anomalies; they are becoming a defining feature of coastal climates. For sessile, habitat‐forming plants such as seagrasses, these extremes can mean the difference between lush, carbon‐rich meadows and bare, eroding sediments. In an article published in this issue of *New Phytologist* (‘Seagrasses Are Most Vulnerable to Marine Heatwaves in Tropical Zones: Local‐Scale and Broad Climatic‐Zone Variation in Thermal Tolerances’), Said *et al*. (2026; pp. 2835–2851) take an important step towards predicting which seagrass species and populations are most at risk, and why. By combining whole‐plant photosynthesis–temperature curves with realistic climate scenarios across Western Australia, they show that vulnerability to marine heatwaves is strongly context‐dependent and often greatest for populations growing in already warm, tropical environments.

*By turning thermal performance curves into maps of risk, Said* et al. *show how physiology can be placed at the centre of climate‐smart management for coastal foundation species*.

At the heart of their study is a simple but powerful question: how close are seagrass populations currently living to their thermal comfort zone? And, how much ‘headroom’ do they have before performance declines? To answer this, Said *et al*. measured net photosynthesis for six seagrass species representing colonising, opportunistic and persistent life‐history strategies, across a wide temperature range (15–42°C) from temperate to tropical Western Australia. They then fitted thermal performance curves to estimate two key physiological thresholds: the optimum temperature for photosynthesis (Topt) and the critical maximum temperature at which photosynthesis drops to zero (CTmax). Several clear patterns emerge.

First, Topt differs markedly among species, spanning almost 10°C. This interspecific variation matches expectations from their realised thermal niches and life‐history traits: smaller, fast‐growing colonisers such as *Halophila ovalis* tend to operate at warmer optima than large, persistent species such as *Posidonia* spp., which are built for resistance but slow to recover from disturbance. Second, even within a species, Topt can vary by up to 4°C across both broad (hundreds of kilometres) and local (tens of kilometres) spatial scales, without a simple monotonic trend along latitude. In other words, a meadow's thermal performance cannot be inferred solely from its position on a map, local environmental conditions and population‐level history matter.

A central conceptual contribution of the paper is the emphasis on Topt rather than CTmax as the more ecologically informative metric for climate risk (Kingsolver & Buckley, 2017). CTmax typically lies well above temperatures normally experienced in the field and reflects an acute lethal limit, relevant for very short, extreme exposures (Kingsolver & Buckley, 2017). By contrast, Topt describes the temperature at which daily net photosynthesis is maximised, and productivity declines on either side (Collier *et al*., 2017). Said *et al*. argue, and demonstrate, that under prolonged marine heatwaves, which persist for weeks to months, it is exceedance of Topt, not CTmax, that signals biologically meaningful stress: reduced carbon gain, impaired growth and ultimately increased susceptibility to mortality (Breshears *et al*., 2021). To make this argument quantitative, the authors take an important step in scaling from instantaneous rates to daily metabolism, following earlier work that derived daily carbon balance by integrating photosynthesis and respiration over natural light–dark cycles (Lee *et al*., 2007). They integrate their experimentally derived relationships between temperature, photosynthesis and respiration with realistic daylengths for temperate, subtropical and tropical zones, in both summer and winter. This allows them to compute ‘daily net photosynthesis’ curves (NPdaily) for each species–location combination and to derive Topt and CTmax for whole‐day carbon balance under different seasonal light regimes analogous to how diel ecosystem metabolism has been used to characterise seagrass responses to warming at the community scale (Burkholz *et al*., 2019). The result is a more ecologically grounded view of thermal performance, not just how leaves respond in a chamber but how entire plants fare over full day–night cycles in different parts of their range.

The final step is perhaps the most compelling from a conservation and management perspective. Said *et al*. overlay their thermal performance envelopes with present‐day and projected temperature regimes for each climatic zone, including realistic marine heatwave anomalies (Oliver *et al*., 2018; Smale *et al*., 2019). This synthesis leads to a striking conclusion: Although tropical seagrass populations often have higher absolute thermal tolerances, they already live closer to their optima, leaving little safety margin (Sentinella *et al*., 2020). Even modest temperature anomalies during marine heatwaves are therefore predicted to push daily net photosynthesis well beyond Topt for extended periods, turning productive meadows into net carbon sources (Collier *et al*., 2012; Burkholz *et al*., 2019). By contrast, some temperate populations have more ‘thermal slack’ and may initially benefit from small warming increments before crossing their own performance thresholds. Overall, the work suggests that five of the six species assessed are likely to experience negative consequences under future marine heatwaves, with the greatest impacts concentrated in tropical regions. By turning thermal performance curves into maps of risk, Said *et al*. show how physiology can be placed at the centre of climate‐smart management for coastal foundation species.

The implications of this framework extend well beyond seagrasses. Thermal performance curves have a long history in both marine and terrestrial plant ecology (Huey & Stevenson, 1979; Berry & Bjorkman, 1980), yet they are still rarely integrated with spatially explicit climate data to inform conservation decisions (Oliver *et al*., 2018; Smale *et al*., 2019). The approach used by Said *et al*. is conceptually straightforward and transferable: quantify thermal performance across species and populations, identify where realised environmental temperatures sit relative to physiological optima and use this information to highlight ‘hotspots’ of vulnerability and refugia of resilience. Similar workflows could be applied to mangroves, macroalgae, freshwater macrophytes or even terrestrial woody plants, especially where long‐lived, habitat‐forming species underpin ecosystem function and carbon storage (Collier *et al*., 2012; Burkholz *et al*., 2019).

The study also speaks to a broader, cross‐disciplinary conversation about plasticity, local adaptation and the scales at which we manage climate risk (Savolainen *et al*., 2013). The mixed patterns in Topt across latitudes and localities imply that both acclimation and genetic differentiation may contribute to observed variation, but these cannot be untangled from physiology alone (Savolainen *et al*., 2013). For evolutionary biologists and quantitative geneticists, the paper offers a clear set of candidate species and populations in which to examine the genomic and epigenetic bases of thermal tolerance. For restoration practitioners, the message is equally salient: ‘local is best’ may not always be the safest rule of thumb in a rapidly warming ocean (Aitken & Whitlock, 2013). Instead, assisted gene flow or climate‐informed seed sourcing – moving propagules among populations that differ in thermal performance – could be explored as tools to enhance resilience, provided that trade‐offs with other traits and local ecological interactions are carefully considered (Aitken & Whitlock, 2013).

There are, of course, important challenges and opportunities ahead. One is temporal; the experiments by Said *et al*. are necessarily short‐term and conducted on adult plants. Yet climate change will play out over decades, and many seagrass meadows are demographically structured, with seedlings, shoots and clonal fragments potentially differing in their sensitivities (Marbà & Duarte, 1998). Another is environmental complexity; temperature does not act in isolation. Light, nutrients, salinity, water clarity and herbivory all modulate seagrass performance and may alter both the position and shape of thermal performance curves (Collier *et al*., 2012; Maxwell *et al*., 2017). Integrating multistress experiments, long‐term field observations and remote‐sensing data with the physiological framework presented here will be crucial for refining predictions (Hossain *et al*., 2015).

A further opportunity lies in linking plant‐level responses to ecosystem‐level consequences. Seagrass meadows are globally significant carbon sinks and biodiversity hotspots (Maxwell *et al*., 2017; Burkholz *et al*., 2019). Shifts in their productivity and persistence cascade to fisheries, sediment dynamics and blue‐carbon budgets (Smale *et al*., 2019). By quantifying how close different meadows are to their thermal ‘tipping points’, we can begin to ask where management interventions such as reducing local stressors, protecting cooler refugia or prioritising certain populations for restoration will yield the largest returns for climate mitigation and adaptation (Hossain *et al*., 2015; Maxwell *et al*., 2017). The Western Australian context of this study, a recognised hotspot for rapid ocean warming and extreme events, makes the findings immediately relevant to coastal managers in the area, but the conceptual template is universal.

For readers less familiar with marine systems, Said *et al*.'s paper also offers a useful point of comparison with terrestrial plant studies. Many of the themes will resonate: the importance of understanding both interspecific and intraspecific variation in thermal tolerance, the difference between survival thresholds and performance optima and the need to consider how daily and seasonal light regimes interact with temperature to determine net carbon gain. Translating these ideas across systems can stimulate fruitful crosstalk between marine and terrestrial plant scientists, ecophysiologists and conservation practitioners. In this sense, Said *et al*.'s study not only sharpens our understanding of seagrass vulnerability but also offers a broadly applicable template for linking plant thermal physiology to climate adaptation strategies across ecosystems.

## Disclaimer

The New Phytologist Foundation remains neutral with regard to jurisdictional claims in maps and in any institutional affiliations.
